# Psychic stress in cranial–cerebral tumors

**Published:** 2009-11-25

**Authors:** V Rotarescu, AV Ciurea

**Affiliations:** ** ‘Carol Davila’ University of Medicine and Pharmacy BucharestRomania; * ‘Bagdasar–Arseni’ Clinical Emergency Hospital,BucharestRomania

## Abstract

From a historical point of view, medicine (in modern society) has set its mission as to cure diseases–medical–type issues–and to ignore patient's condition–illness endurance. By accepting this point of view with reference to their problem, patients have entered some sort of conspiracy of silence and started ignoring the emotional reaction to their medical problems (or to cancel these reactions), regarding them as irrelevant to the problem itself. This approach is also reinforced by the medical pattern that absolutely contests the idea that mind influences the body in a very important way.

Another equally unproductive ideology is the idea that people could cure themselves alone even of the most serious illnesses by feeling happy or by thinking in a positive manner, or the idea that people are guilty of getting sick. This attitude resulted in creating a widespread confusion and in significant misunderstandings regarding the extent to which illness can be influenced by mind, at times even blaming someone for having got ill.

In the world of illness, emotions have supremacy and fear is the only ‘thought’. We can be so fragile emotionally speaking when we suffer from some illness because part of our mental good–humor is partially based on the illusion of invulnerability. Illness–especially a severe one – destroys this illusion and cancels the premise that our world is one in which existence is completely secure. All of a sudden, we feel thick with weaknesses, we feel helpless and vulnerable [12].

There is a problem when the medical personnel ignore the way patients react from an emotional point of view, even though they should exclusively take care of our physical state. This lack of interest in the emotional reality of an illness neglects something obvious which shows that the emotional state of people can play a significant role as to their vulnerability towards illness and during the recovery process [4].

There is already a scientific basis according to which a limit to the medical efficiency appears not only during the prophylactic process but also during treatment. This efficiency can be enhanced by curing the emotional state of the patient at the same time with his/her medical state. Unfortunately, too often, the medical personnel is in a hurry or unsympathetic towards patients' despair and, seemingly, things get worse indeed in the midst of the medical system harsh reality, medical system that depends too much on time keeping performed by accountants [5].

## Cranial–Cerebral Tumors (CCT)

All cells in a body are structured following the same pattern: they have a nucleus that contains chromosomes and genetic material with chains of DNA, a gelatinous cytoplasm with mitochondria to create energy and ribosome for protein synthesis. Each cell in a body has an identical number of chromosomes, 46 for a human. In addition to the fact that they have the characteristics of a body system and it controls the transfer traits from one generation to another, chromosomes and DNA manage the very cell that contains them. In spite of the fact that cells have the same basic pattern, they inexplicably specialize during the development of the body system. In reference to the nervous system, we notice that this one owns a discrete, discontinuous structure. From a histological point of view, the nervous system consists of cellular elements, representing 75% of the entire mass of the nervous tissue (35% neuronal cells and 40% neuroglial cells), of intermediary non–cellular substance (extra–cellular fluid with low–molecular elements) that represents 15%, and of the blood vessels network that occupies 10%. These proportions vary significantly depending on the nervous tissue characteristic: white substance or grey substance, central segments etc. (9, 10).

The key to the nervous system strategies is based on subdivision and on functions location. In the brain, the specific aspects of the informational processing are limited to certain specific regions. For instance, each sensorial possibility is processed in a distinct region sensorial connections are precisely represented, in the shape of a map of the corresponding body area. Muscles and movements are also represented in an ordered sequence of connections. Thus, the brain contains at least two types of maps: a type for the sensorial perceptions and another one for the motor commands [2]. Nervous cells differ from the other cells of the body in that they can communicate rapidly ones with the others, sometimes at great distances but very accurately. This rapid and accurate communication is possible due to the signaling mechanisms–axonal conduction and synaptic conduction [22].

In spite of the anatomic differences–micro and macroscopic, the nervous system constitutes a unitary functional mechanism, that completes one type of activity–reflexive activity–as a physiologic or psychic response (physiologically mediated) to the stimuli in the interior or exterior world of the body system. Hence, we cannot talk about neuronal formations having exclusively physiologic function or exclusively psychic function. To understand this behavior, we need to consider the highly complex way the nervous system is functionally and anatomically organized [2].

## Circumstances that Trigger the Occurrence and Development Of the Cranial–Cerebral Tumors (CCT)

The modern oncology data show that the malign process occurs on those tissues that have previously undergone certain pathological mutations: inflammatory, proliferative, dystrophic, irritant, and traumatic. What characterizes these preliminary lesions is the fact that they do not tend to regress, but they form ‘pre–cancerous lesions’. Exogenous and endogenous factors, of general or local kind, radically intervene and modify the type of metabolism of the said tissues, modifying their biochemical and cellular structure, causing metabolic and tumor structural characteristics in them, characteristics that are called cancerous factors. Still, the clinical remarks show that, in numerous cases, these two conditions (pre–cancerous lesions and cancerous factors) are not sufficient for the intracranial tumors to clinically manifest. These may be latent for a long time and only the occurrence of a third kind of triggering factors will cause the clinical phase accompanied by all characteristic symptoms to happen [10].

*Incidence of cerebral tumors*, versus the other tumor diseases of the body system, is estimated to correspond to 8% out of which 25% represent cerebral metastases as a percentage that, lately, has been increasing continuously. In children, cranial–cerebral tumors represent the second malignity cause after leukemia [9, 5]. Once the CT–scan and the MRI have been established as a routine check, cranial– cerebral tumors can be diagnosed more rapidly and more accurately as far as the histopathology specificities are concerned but their incidence impressively increased in the last 15 years, in all ages, twice more frequent in men.

*Location* is approximately 2/3 supratentorial (in order of frequency: frontal, temporal, parietal, intra– ventricular, and of basal nuclei) and of 1/3 subtentorial with predominance in children (70%).

*The cranial–cerebral tumors symptomatology* depends on age, location and their nature; it can fell into two significant groups:

1. Symptoms caused by the increase in the intracranial pressure: headache, throwing–up, papillary edema.
2. Symptoms caused by the lesion location and constituted by the multitude of neurological, irritant and/or defective signs.

*The starting point and the evolution* are usually slow, progressive, gradually building up neurological signs that are revealing for a cerebral location, against a clinical background of intracranial hypertension. Tumors can appear all of a sudden only in the case of intra–tumor bleedings or in the case of intra–tumor cists occurrence. The high tolerance manifested by the nervous system as far as their appearance is concerned causes the neurological focal syndrome to be minimum. If they are not diagnosed at the beginning, surgical treatment will be delayed and the continuous evolution will lead to a gradual recurrence of the CNS structures, the neurological deficit irreversibly increasing (plegia, aphasia, blindness, deafness etc.) and causing the self–consciousness state to turn from sleepiness to coma.

Prognosis is influenced by the histological structure of tumor, most of the times in a gray manner since the majority are neuroectodermal tumors (75%) and some of them manifest an increased malignity or can be extremely obtrusive; as far as the median tumors are concerned, surgical accessibility is difficult.

*Treatment* of CCT is a complex one if a complete recovery from these serious lesions is to be attained.

1. *Surgical*–since tumors are processes that replace spaces, they endanger directly a patient's life not only by their histopathological nature but also by their presence in the brain cavity. Surgery must start from certain principles: complete abscission, causing no injuries to the adjacent vascular–nervous structures and recovering the circulation ways of the cephalo–rachidian fluid (CRF). Recent data show that mortality strictly intra–surgical is less than 3%. The principle of complete abscission cannot be always attained because of the surgical technical difficulties; in this case, partial abscissions being targeted in order to prolong or save a patient's life. Sometimes, surgical attitude must be limited to palliative interventions that will solve only intracranial hypertension (draining of the cephalo– rachidian fluid), while radiotherapy will be used in order to stop the tumor from growing. Stereotaxic biopsy has been greatly used in the last decade for the histological type of tumor to be established, being followed by surgical removal by means of craniotomy or radiotherapy and guided by CT–scan or MRI.
2. *Radiotherapy* has a favorable effect on radio–sensitive tumors such as the medulloblastoma, ependymoma.
3. *Cytostatic chemotherapy* is helpful in CCT grade Ⅲ,Ⅳafter the surgical and radiotherapy treatment had been applied, in order to avoid recurrences and dissemination thru CRL.
4. *Corticotherapy *reduces brain metabolism of glucose.

The results of multi–way treatment in CCT must be observed continuously both clinically (ex. Neurological) and paraclinically (CT–scan, repeated NMR) accompanied by a general haematological and biological check. Healing is considered after 10 years of following–up.

*Recurrences* call for another mandatory surgical intervention. From the beginning, one must differentiate the actual tumor growing recurrences of the subtotal ablated tumor by means of CT–scan and repeated MRI which evince tumor reappearance before the neurological signs show up. A new intervention in malign gliomas depends on many factors: histopathological structure, patient's age, his/her clinical condition (determined by the Karnofsky score).

*TIC metastasis*. Actual cerebral tumors do not cause metastasis. Gliomas can attain, after surgery, a regional invasion thru contiguity. Some cerebral tumors (ependymoma, pinealoma, malign astrocytoma) can be spontaneously introduced by liquid way by means of tumor fragments detached after cranial–cerebral trauma or after surgeries for tumor ablation. Neoplastic cells are frequently introduced even opposite the CRL circulation, as a result of the presence of the tumor, to which regular physical efforts (even the respiratory movements) or those of the surgical intervention kind or the hypertonic substances infusion are being added.

The exceptional diversity of cerebral lesions, as well as the great severity of motor, sensorial and psychic difficulties that they drag along, calls for a most immediate and most accurate diagnosis so that the chance of survival of the minor lesions patients to increase.

## Psychic Disorders in Cranial–Cerebral Tumors (CCT)

The interest shown as to the psychic disorders manifested in cerebral tumors is very important, although it is said that the possibility for a cerebral tumor to cause psychic distresses in a patient is rarer for a psychiatrist than for a neurologist. If many patients suffering from cerebral tumors often show, during their development, noticeable psychic disorders–from 10% up to 100%–depending on the attention paid while they are reached and recorded, as well as on the development stadium of the tumors at the moment of the examination, there are also cerebral tumors with the patients suffering from psychic illnesses, meningiomas and gliomas being the most frequent types.

During the development of the cerebral tumors, psychic disorders are characterized by a start and by a clinical picture that depends on the nature, location and developmental behavior of the tumor; they are accompanied by neurological signs in the shape of a global cerebral distress or of a focal syndrome, that can point to the tumor's location.

As far as the etiopathogeny is concerned, no connections between the tumor material and the psychic phenomenology could be established, the evolutionist character of the lesion impeding the possibility of establishing precise clinical–pathological correlations. Pre–morbid personality as well as predisposition of the field seems to play a major role. There are no precise data as to the way changes in the cephalo–rachidian fluid, circulatory changes, global distress through ICH (IntraCranial Hypertension) influence psyche differentially in proportion to direct effects of tumor lesion. The overall distress of the brain, due to tumor toxic effects or ICH, causes general psychical disorders, and the consequences for tumor location trigger psychical focal disorders (moria, the Gerstmann syndrome, spatial agnosia etc.)[11].

Bagdasar and Arseni (1951) noticed, as far as the frontal tumors are concerned, in case the tumor is small and it grows in the very cerebral parenchyma, an exaggeration in premorbid personality along with central, discrete, contralateral facial paresis. Moria occurs only when, as a result of growing, tumor compresses the frontal lobe on the opposite side, revealing traits that are opposed to the premorbid personality (accompanied by a contralateral hemiparesis). When the basal nuclei are compressed, the confusion state, somnolence, and gatism manifest (contralateral hemiparesis and hemiplegia, signs of pyramidal ipsilateral inflammation). Aggressiveness, restlessness are also specific signs of frontal locations.

Positive diagnosis is determined by identifying:

1. *A narrowing in consciousness field*, given in 1/3 of cases, initially a slight narrowing, while a confusion or confusion–oneiric state of different intensities subsequently develops.
2. *Apathetic state*, based on which irritability and a pronounced emotional lability show up, and which is not always explained psychologically; euphoria rarely occurs, while sadness signs are more frequent (it differs from depression).
3. *A gradual deterioration of intellectual functions *: decline in the capacity for abstraction, synthesis and analysis, bradypsychia, failure of correlations, leading towards the chronic psycho–organic syndrome (the risk of a false diagnosis with senile or arteriopathic dementia).
4. *The delirious syndrome*, slightly systematized, is more rarely found but acute psychotic episodes are also likely to occur.
5. *Hallucinatory seizures*: visual, auditory, olfactory, sometimes taking the shape of an aura or an equivalence of a comitial seizure.
6. *Paroxystic alteration in consciousness*: uncinate seizures with ‘dreamy state’ or ‘absences’ with motor automatisms.
7. Seizures of confusion state worsening, up to coma.
8. Secondary comitial seizures.

*Intracranial hypertension (ICH) syndrome* can occur before or after the focal signs. Usually, it initially manifests remission episodes, but it gradually becomes permanent, only to end in coma and exitus. It includes:

1. *Somatic symptoms:* headaches, nausea, vomiting, papillary edema, bradycardia and bradypnea, generalized convulsive seizures.
2. *Psychic symptoms:* mental confusion and dysmnesia. Mental confusion involves alertness and psychic rhythm, due to a diencephalic touch and it less affects the elementary intellectual functions. Dysmnesia is given in 1/3 of cases, it is more frequent in elderly persons and it may get to the Korsakov syndrome itself; allegedly, it is due to damage through ICH of nervous centers located in the third ventricle's floor.

They mention the positiveness of the neurological exam in 83% of patients considered ‘psychic’ persons having, in reality, cerebral tumors, which calls attention to the necessity of undergoing a thorough neurological exam, with use of modern means of diagnosis (cerebral CT, cerebral NMR, CT–scan etc) [8]. Observing patients after tumor ablation usually showed psychic disorders relief. After–surgery sequels may occur in the shape of over–added psychic disorders in a patient that did not manifest them or vaguely manifested them prior to surgery (for instance, paranoid delirium, atypical depressive condition). In corpus callosum tumors, psychic disorders have a 100% incidence, with a characteristic aprosexia. Subtentorial tumors manifest an early ICH, and they are characterized by paroxystic anguish seizures. Tumors located at the brain base are not related to characteristic psychic disorders

**Table 1:** Clinical Chart of Focal Psychic and Neurological Disorders

Location	Psychic Disorders	Neurological Disorders
Frontal Tumors	Moria (dumb, puerile, artificial euphoria condition against a general apathy, inadequate jokes, puns); General activity reduced up to akinesia or aggressiveness and agitation;	Paralyses (monoparesis, monoplegia,hemiparesis, hemiplegia); Reflexes disorders: occurrence of grasping and grabing reflex; aversive, oculocephalic seizures; frontal ataxia;language disorders; aphemia.
Temporal Tumors	Temporal Epilepsy: epileptic aura; psychical equivalence in psycho–sensory seizures in the shape of visual, auditory, olfactory, gustatory, vestibular hallucinations (stereotypically repeated and against a confusion state; visual hallucinations are projected on the visual field opposite the tumor, with homonymous hemianopsia); motor seizures: minor automatisms (splashing, mastication, swallowing) or major automatisms (run, ambulatory automatism); consciousness disorders; uncinate seizure (dreamy state, unpleasant olfactory and gustatory hallucinations, ‘deje–vu’ paramnesic disorders; vegetative seizures: painful epigastric and abdominal seizures, vasomotor and respiratory disorders.	Homonymous hemianopsia on the superior side; Aphasic disorders of sensorial type
Parietal Tumors	Common Psychic Disorders	Body schema disorders: Gertsmann syndrome (digital agnosia, acalculia, agraphia, right–left disorientation); Anton–Babinski syndrome (hemiasomatognosia, anosognosia, anosodiaphoria);Contralateral sensibility disorders(tactile astereognosia agnosia, profound and tactile hemihipoestesia);Motor disorders (discrete hemiparesis or hemiplegia);Muscle contralateral atrophies; Ideomotor apraxia; Sensorial aphasia (in lesions of the dominant hemisphere)
Occipital Tumors	Elementary visual hallucinations (they reoccur stereotypically and they can precede a convulsive seizure)	visual agnosia; cortical blindness; contralateral homonymous hemianopsia

## Psychic Stress

### Common Facts

It has been a long time since Epictet (a stoic philosopher, approx. 50–138 A.D.) noticed that: ‘things are not the ones that trouble people, but the ideas they have of things’, a statement that anticipated the essence of contemporary research. Present data in the specialized literature underlines the coexistence of various ways of defining and understanding stress [16].

The fact that, after 50 years, the stress notion continues to be operative proves the impact this concept has on science progress, materialized in the huge volume of data and studies in the most varied fields: biology, medicine, psychology, sociology, philosophy, ecology, etiology, gerontology. It is ascertained the proliferation of publications on stress, estimated to reach 120 000 till 1981, the issuance of new magazines as well as the publishing of a great number of monographies.

The need to improve the quality of life, to reduce mortality and morbidity, promoted interdisciplinary sciences, such as behavioral medicine, whereas medical fields together with the psychological ones try to explain human adaptability as well as health and illness mechanisms. Within the contemporary science context, it is ascertained the fact that there are researchers that agree with H. Selye's ideas, some of them using modified versions of his ideas, others regarding them as not verified work hypothesis while other researchers reject or ignore them. But there are few those researchers that still use the term stress according to Selye.

Stress theory must be regarded as a concept that is open to new assimilations and acquisitions, able to originate new horizons in research on the complexity of the human– environment interrelation complexity and new progresses in learning certain psycho–biological processes such as adaptability, homeostasis or even ageing.

## The Concept of Psychic Stress

The *homeostasis* concept, foreshadowed by Claude Bernard (between 1870–1880), naturalized by Cannon (1932), enhanced by Henry Selye (1936), has been deeply rooted by modern biology, including in point of cybernetics. Wittenberger shows that homeostasis is an essential feature of biology and of life on the whole and that the homeostatic functions of balance– stability, and of optimization–adaptability, also condition the organism autonomy process in regard to the environment and its active intervention on environment. In certain author's points of view [17] who drafted the cognitive theory of psychic stress, this one is caused by a discrepancy between a person's resources, abilities, capabilities and the requests enforced on him/her.

Mihai Golu defines psychic stress as: ‘a tension, strain and discomfort state caused by affectable agents with negative significance, resulted from frustration or suppression of certain motivational states, from the difficulty or impossibility of solving certain problems’ [4].

*It is said that psychic stress has a primary attribute when it is the result of an attack perceived in the psyche area and a secondary attribute, this one representing in fact a supplemental reaction or an awareness reaction regarding a biological, physical etc. stress, which is endowed with a threatening significance.*

Looking through the specialized literature (1; 6; 7; 3; 19; 20; 27; 14; 25, 26), one can state that, in order to get settled, psychic stress involves perception, acknowledgement and interpretation by the human individual, in a manner that is strictly particularized and catastrophic, requesting a mandatory interaction with a stimulus in a social context. Hence, we get to the occurrence of important interindividual differences in reactions to stressful situations, the irrelevance of interrelating measuring psychological criteria with various physiological signs of stress as well as the occurrence of clear differences between natural conditions and the laboratory ones in generating stress.

### Vulnerability to Stress

Lazarus [18] considers that the effects of the stressing agent depend not only on its own characteristics but also on two attributes belonging to the receiving subject personality, namely: *the quality of the emotional answers and the adaptability strategies put to work.*

Vulnerability to stress is recognized as a feature proper to certain individuals of facilely reacting through a psychic stress to a large variety of stressing agents.

### Adaptative Strategies

Psychic stress primarily includes among its manifestations psychic symptoms, as well as behavioral manifestations. Likewise, any psychic phenomenon, externalized or not (from the cognitive processes and up to the volitional and emotional processes) is accompanied by hyper or hypofunction physiological phenomena of the internal organs, neurohumoral–mediated. [13; 15) The most known somatic changes, induced by the evolution of some psychic processes, are the so called physiological correlations (somato–visceral) of emotions: tachycardia, muscular tone disorders, secretory disorders etc., materialized in expressions like: ‘my heart is beating hard’; ‘my heart stopped’; ‘he/she turned white with fear’; ‘he/she got a lump in his/her throat’ etc.

Cognitive theories of emotions speak of three types of human reactions to any threat to his/her harmony and interior equilibrium: fear, shame and guilt, set off by his/her own comments or by the comments of the people around, sometimes rapidly overcomed, while other times continuing as long as the threat persists.

Motivational theories of stress, having explanatory roots in Sigmund Freud's theories on frustration, as well as on Maslow's theory on motivation, explain stress starting from the impossibility, in a circumscribed moment in time, of satisfying an important motivation. This would trigger, without effort or without becoming aware, the defense mechanisms of the human psyche (R. White, 1952).

Any unfulfillment of a goal, even a minor one, originates a f rustration which triggers compensation phenomena. This strive, for compensation or recovery, burns psychic energy (if it is a conscious process), physical or psychosomatic energy, (if they occur on the subconscious level)–according to psychologist R. Lazarus [18]. The maximum level the individual aims at is that of adjusting to the social environment requirements, using for this reason various types of adaptative strategies.[16]

As a consequence of the environmental stable factors as well as of the personality ones, the issue of interindividual differences explains the varied ways of facing / confronting constant stress sources. The complexity of this phenomenon that intersects the stressing agents and the emergence circumstances explains human variability regarding the use of adaptative strategies. The vulnerability to stress, regarded as a personality trait, can be reduced by a positive approach to stress. (23; 21; 6)

### Psychic Stress in CCT

Unlike the mechanism of a biological function that manifests a single level attribute and it produces the said function automatically, the mechanism of the psychic function has a multilevel structure and it necessarily produces the said function, by receiving and interpreting the outside informational signals. Likewise, as part of a very orderly integrative system, the state of a concrete psychic function will essentially depend on the state of the other system components. This particularity at the psychic functions level will also be reflected in the neuronal mechanisms.

*At the brain level, the configuration of mechanisms that are specific to the various psychic functions runs according to systemic principles*. Consequently, we will not have to deal with isolated, closed, individual mechanisms, but with interdependent mechanisms, that influence and condition each other. Besides the neuronal structures themselves and the connections between them, psychic strings must also be added to the mechanism of a psychic function. The perception mechanism includes: the influence prompted by language, the mnesic string, the influence of thought, and the emotional–motivational string; the mechanism of thought in its turn includes: the verbal string, the perceptive string, the mnesic string etc. Therefore, it is obvious that the involvement of the psychic strings institutes significant changes in the functional state itself of the neuronal structures that are part of the primary mechanism of the process taken into account. The events development logic in these structures will depend not only on the present but also on the past, the mechanism of psychic operation includes spatial as well as temporal coordinates and, along with the substantially– energetic component, it also comprises informational components. *In other words, the neuropsychic mechanism is a logistic, auto–adjustable and auto–organizing structure.*

The human brain develops a form of psyche, of a socio– cultural nature, as a result of the individual interaction and communication with the socio–cultural environment. The psyche is developed as a receiving, processing, interpreting and storage function of the information provided both from outside and from inside the organism. The information is constituted through successive processing and integrations of the brain in relatively distinct entities that we call psychic processes and states. Any psychic process is subject to the command and control principle, mediating and adjusting the dynamics in the individual's relation with the external world.

The human being has the biggest capacity to adapt backed up by an extraordinary growth in the psychic functions and processes, especially the awareness ones

As to the pathologic deteriorations, alterations in consciousness intertwine with alterations in thought and vice–versa. The integrative hierarchical levels arise and interpenetrate in a unitary system and, between the consciously organized level and the unconsciously organized one; there is a constant interaction and interconditioning with concordance and discordance relations (conflict). Thus, each behavioral act is realized based on mediation both of conscious and of unconscious that communicate and constantly interpenetrate each other.

The consciousness mechanisms play a dominantly controller role on the unconscious ones. Preponderantly structuring itself on the primary biological motivation, the unconscious is, dynamically speaking, the result of impulse–reaction sequences, direct and relatively constant links, between stimuli and answering vegetative–motor reactions, of the unconditional response type. In the human being, such behaviors can be noticed only in affect states (emotional explosion), in somnambulism states or in ebriety states whereas the control of reason, of conscious is heavily diminished or completely suspended.

As to the normal daily behavior, the unconscious elements, mostly energetic ones, are incorporated into patterns that are elaborated and controlled at the conscious level. This way, the unconscious influences and modulates the dynamics of the conscious structures, and the conscious exerts its controller influence through analysis and critical evaluation of the unconscious contents sending messages of temperance, repression, postponing, transformation, ranking etc. [24].

The unity of the human psychic is not plane and inert. Due to the structural and functional heterogeneity of components, besides the consonance relations there may also be contradictions, divergences, disharmonies related with a greater or smaller degree of tension subjectively lived in various shapes: restlessness, discomfort, bad mood, irritation, indecision, obsession, emotional bewilderness, motivational conflict, etc. All these give to human's behavior and existential condition a scenical note, more or less emphasized.

The intensity of intrapsychic tensions and dissonances depend not only on the exterior environment influences but also on the particularities of the psycho–physiological structure of the individual. In general, tension and frustration act as progress and evolution factors of the personality system, triggering specific exploration–investigation components, of learning, of creating new procedures and modalities of confronting and acting. But, when the intensity and duration of these states cross certain valoric limits, they turn from optimizing factors into disturbing agents, that generate oscillations with pathological character.

Due to their disturbing effects, the dysfunctions at the psychic system level negatively reflect on the brain condition (metabolism changes, circulatory changes, irritability changes) and through the brain they negatively reflect on the biological/physiological state of the organism on the whole. This way the somato–psycho–somatic circuit closes, and this triggers, in fact, the unity of the personality system.

Playing the role of a specialized controller organism in the body and in the individual relation with the environment, the brain has as an intrinsic function the gathering, processing and using of information. In its highest form of expression, processed and integrated information is represented by psychic procesuality that opens between the profound unconscious and the acutest conscious. Being of informational essence, the psychic cannot take shape and manifest itself as a specific reality but in the brain communication relation with the signals sources in the outside and inside environment of the organism [24]. Unlike the biological information whose identity lays over the biophysical and biochemical transformations and whose mechanism is actually constituted by the entire organism, the psychic information tends to detach itself and individualize itself in relation to the substantially–energetic codes that it uses to transmit itself and it occurs only as a result of certain specialized mechanisms–neuronal structures. Thus, the quality differentiation degree inside the psychic information, its structural complexity, and its controller value will be decisively conditioned by the evolution–structuring and integrity degree of the nervous system. The involvement of hormones in the physical and psychical stress reaction cannot break off from the one of CNS and autonomous due to the complexity and interrelation of the three systems activity. This aspect is valid and refers to the immune system, the neuroendocrine system having close connections with it.

Cancer continues to be regarded as a more serious illness than any other. The illness occurrence calls for new requests, operates important and inherent changes in the patient's mood. Balzac reckoned long before Selye: ‘…fear is a feeling that gets you ill half way and that violently attacks the human vehicle so that its capacities are pushed either to the greatest degree of their force or to the lowest degree of deterioration.’

Besides the two big issues of the diagnostic – the idea that they suffer from a disease that put their life in danger and the treatments that are difficult to bear – patients suffering from cranio–cerebral cancer also have to face the location issue. The brain has the most important significance related to the autonomy essential need and that is why the patient is filled with a severe insecurity, confusion as to the future, feeling devastated and desolated. The picture of the psychic states that usually accompanies cancer (fear of illness, of relapse, of suffering, of death) is combined with a large range of sufferings specific to cerebral deteriorations triggered by the lesion location and this prompts a fearful feeling of helplessness with a strong feeling of inadaptability.

Unlike the reactions of those with tumors located some place else, for patients with cerebral tumors finding out their diagnosis is an emotional catastrophe up to shock, that is amplified by surgery perspective. Right after surgery, concerned with physically getting fit, patients have neither time nor psychic energy to reflect on long–term repercussions and they may feel relieved, sometimes even euphoric. But, when they get back home, although physic recovery is good, they are psychically dominated by fears regarding treatments that will follow, regarding their result as well as fear of relapse.

The personal resources, the inner force, the reaction way (coping with) are very important for the chosen strategy and for the patient' behaviors in dealing with an illness. Providing, with sensibility and without haste, clear and precise information may help the patient while being given the news and it may also help him find something that will give him hope.

The patient is usually fighting fear of diagnosis and being overloaded with information, which cause anxiety, can compromise his/her capacity of making a decision. It is good to know the amount of information he wants to know and the amount he is able to receive, giving the unusual situation he/she is in (attention disorders, memory disorders, understanding disorders etc.); some patients only wait for encouragements. The main objective must be the one of helping him/her find the effective answer so he/she can adapt to the situation. The true autonomy and the respect for the patient should consider some patients' wish of declining his/her doctor's responsibility, situation that is called ‘psychological autonomy’, which means that the patient that has been informed decides not to make a decision himself/herself but his/her personal doctor.

It is well known the effect of a positive emotional mood on the immune controller and endocrine systems. Such attributes as: hope, optimism, joy, vigor, strength, force, have been currently associated to the response to illness, the phenomenon of the psycho–social support triggering a positive mood improvement mechanism or in a series of response patterns, classified as what it is called ‘good mood’.

Various studies revealed the fact that psycho– social intervention enhances not only the patient's comfort but also his/her life quality itself and that it can help him leave longer. The results of these studies clearly indicate the fact that psycho–social interventions have positive effects on emotional adaptability, functional adaptability and on symptoms related to the illness and treatment in the case of patients with cranial–cerebral tumors. 
